# The Ultrastructure of the Nictitating Membrane of the Little Penguin (*Eudyptula minor*, Aves)

**DOI:** 10.1093/iob/obaa048

**Published:** 2021-01-05

**Authors:** S P Collin, H B Collin

**Affiliations:** 1 School of Life Sciences, La Trobe University, Bundoora, VIC 3086, Australia; 2 Oceans Graduate School and the Oceans Institute, The University of Western Australia, Crawley, WA 6009, Australia; 3 Department of Optometry and Vision Science, University of New South Wales, Kensington, NSW 2052, Australia

## Abstract

The ultrastructure of the nictitating membrane in the little penguin *Eudyptula minor* was studied using both scanning and transmission electron microscopy to improve our understanding of the function of ocular adnexa in diving birds. Following euthanasia, eyes were enucleated and immersion fixed in Karnovsky’s fixative. The nictitating membrane and conjunctiva were embedded in araldite and semi- or ultra-thin sections were stained and photographed using compound and transmission electron microscopes, respectively. Ultrastructural dimensions were measured directly from digital photographs. Surface ultrastructure was examined using scanning electron microscopy. The transparent nictitating membrane consists of a dense stroma surrounded by epithelia on both the external (conjunctival) and internal (bulbar) surfaces. The conjunctival surface of the membrane near the leading edge is covered by microvilli, which transition to microplicae and finally to microridges in the periphery. Beneath the epithelial cells, there is a well-developed basement membrane. Scattered throughout this epithelium are a few goblet cells. The surface of the bulbar epithelium is covered by microvilli near the leading edge, which become denser peripherally. The stroma consists of densely-packed collagen fibrils, which are randomly oriented in bundles near the leading edge but are aligned in the same direction parallel with the epithelial and corneal surfaces and with the leading edge, when the membrane is extended. The ultrastructure of the nictitating membrane in the little penguin differs from other birds and its function is predominantly protective, while preserving clear vision in both water and air.

## Introduction

The nictitating membrane is a thin translucent sheet of ocular tissue situated dorso-medial to the globe, which is actively retracted ventro-laterally across the cornea. In vertebrates, this mobile component of the ocular adnexa assists in spreading the tear film across the cornea, thereby removing any debris and acting as a barrier in the protection of the cornea and eye (Sivak and Glover 1986). Nictitating membranes have been described in many vertebrates including sharks (Gruber and Cohen 1978; Poscai et al. 2017; Collin 2018), amphibians (Lande and Zadunaisky 1970), reptiles and birds (Sivak and Glover 1986; Kühnel and Schramm 1989; Schobert et al. 2013), and mammals (Stibbe 1928; Barasa 2003). In birds, the “membrana nictitans” or third eyelid is pulled temporally from the medial canthus of the eye by the pyramidalis muscle located behind the globe (Friedmann 1932; Sivak and Glover 1986). The free (leading) margin of the nictitating membrane (plica marginalis, Baumel 1993) is thought to serve as a lock to help hold the nictitans in place when it is drawn over the eye (Slonaker 1918; Sivak and Glover 1986). Despite early suggestions that the nictitating membrane in diving birds may have a refractive role in helping to compensate for the loss of corneal power in water, this does not appear to be the primary function of the membrane. Since the refractive index of the nictitating membrane and the cornea are similar and the curvature of the membrane closely follows that of the cornea, it is therefore not thought to contribute to the power of the eye (Ischreyt 1914; Sivak et al. 1978).

The morphology of the nictitating membrane has been described in various species of birds (Stibbe 1928; Leeson 1961; Williams and Flach 2003; Schobert et al. 2013; Jochems and Phillips 2015) but, in general terms, the membrane comprises an outer or conjunctival epithelium, a connective tissue stroma, and an inner bulbar epithelium (or endothelium), which is moved by two muscles, the musculus quadratus membranae nictitantis and the musculus pyramidalis membranae nictitantis (Baumel 1993). However, there is only one study which examines the structure of the nictitating membrane in a penguin, namely the rockhopper penguin (*Eudyptes crestatus*) (Sivak and Glover 1986). This study uses both light and transmission electron microscopy and reveals that the membrane is thinner than the underlying cornea and that the stroma is avascular, with an arrangement of collagen lamellae behind the leading edge of the membrane (marginal plait) that is ordered, indicating a heightened level of transparency, which may be useful for underwater vision and predation on their preferred diet of krill, squid, and fish. These sub-Antarctic diving birds usually remain in shallow water, but are capable of diving up to 330 feet in depth.

This study examines the ultrastructure of the nictitating membrane and conjunctiva of another species of penguin, the little penguin *Eudyptula minor* using light microscopy and both scanning and transmission electron microscopy. *Eudyptula minor* are the smallest of all penguin species, consuming about 25% of their body weight of small fish and squid, daily. These little penguins are diurnal, generally forage at or near the surface (Cannell and Cullen 2008) and rely on vision, where the eyes possess high densities of neurons for acute vision across the horizontal meridian of the retina panoramically targeting prey (Coimbra et al. 2012). Although similar to *E. crestatus*, the nictitating membrane of *E. minor* reveals some major differences in the surface and internal ultrastructure, which may support a protective role in how these diving birds navigate their amphibious lifestyle.

## Materials and methods

### Animals

**Fig. 1:**
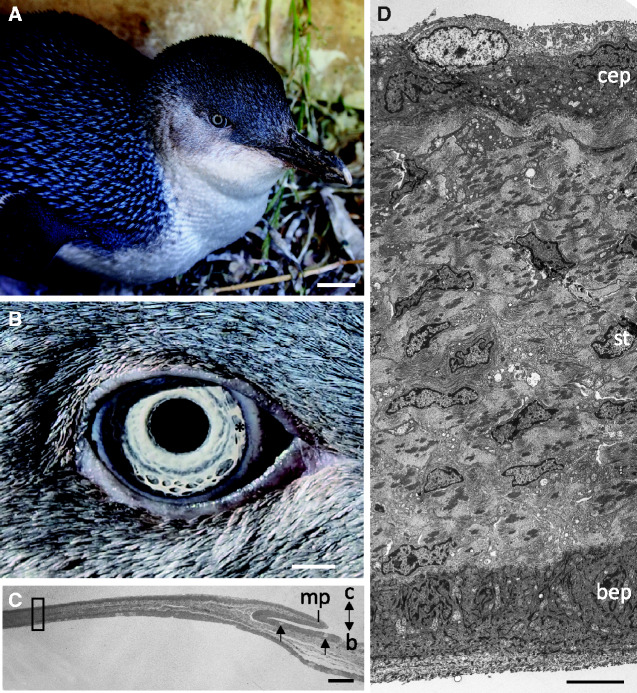
The nictitating membrane of the little penguin *Eudyptula minor*. **A**) Photograph of *E. minor*. **B**) Close up of the right eye of *E. minor* showing the leading edge of the retracted nictitating membrane (*) emanating from the medial canthus. Beak is toward the right. **C**) Light micrograph of the partially extended nictitating membrane. The orientation is conjunctival (c) to the top and bulbar surface (b) to the bottom. The electron micrograph in D is from the region highlighted with a rectangle. Arrows depict goblet cells in the peripheral region of the nictitating membrane. **D**) Electron micrograph of a transverse section of the membrane near the advancing edge showing a dense stroma surrounded by epithelia on both the external (conjunctival) and internal (bulbar) surfaces. bep, bulbar epithelium; cep, conjunctival epithelium; st, stroma. Scale bars: 25 mm (A); 40 mm (B); 50 µm (C); and 50 µm (D). Photographs in A and B courtesy of B. Cannell.

Eyes were obtained from two adult little penguins, *E. minor* (Fig. 1A, B). Little penguin eyes were collected from individuals that had to be euthanized at the Veterinary Hospital at Perth Zoo, Western Australia. All procedures in this investigation were approved by The University of Western Australia Ethics Committees (AEC No. RA/3/100/927).

### Fixation and histological preparation for transmission electron microscopy

Following euthanasia, all eyes were enucleated from the orbit and immersion fixed in half strength Karnovsky’s fixative (2% paraformaldehyde, 2.5% glutaraldehyde, 0.1 M sodium cacodylate buffer, 2% sucrose, and 0.1% calcium chloride at pH 7.2) for 24 h at 4°C, rinsed in 0.1 M sodium cacodylate buffer and post-fixed in 2% osmium tetroxide. The orientation of each eye was noted by ocular landmarks and the anterior segment (cornea and nictitating membrane) was carefully separated from the remainder of the eyecup with care taken to identify both central and peripheral regions for later analysis. The Harderian gland and associated duct were not excised.

Following post-fixation in osmium tetroxide, the cornea, nictitating membrane, and conjunctiva were embedded in araldite and semi- or ultra-thin sections cut on an ultramicrotome (LKB) in the radial (transverse) plane with respect to the optical axis of the eye using glass knives. Selected semi-thin sections were stained with Toluidine blue, photographed using a BH-2 Olympus compound light microscope fitted with a digital camera (Olympus DP30). Dimensions of the nictitating membrane and adjacent conjunctival tissue were measured directly from digital photographs using Image Slave software (Optimas, Adept Electronic Solutions, Australia).

Selected ultra-thin sections were mounted on grids and prepared for transmission electron microscopy by staining with lead citrate and uranyl acetate according to Collin et al. (1999). Sections were examined on a Philips 410 transmission electron microscope and photographed using Kodak Technical Pan black and white film rated at 100 ASA and digitally. All measurements were performed on enlargements of electron micrographs using a magnifier and graticule and are presented as a mean and standard deviation. Data available on request.

### Histological preparation for scanning electron microscopy

Following post-fixation of the anterior segment and adjacent conjunctival tissue in 1% osmium tetroxide in 0.1 M cacodylate buffer and dehydration in a graded series of alcohols, ocular tissue was critical point-dried in a Polaron (Watford, UK) critical point dryer and mounted on 10-mm aluminum stubs with double-sided tape. Each piece of nictitating membrane was oriented and/or hemisected so that half of the tissue piece was inverted and both sides were displayed in order to ensure both epithelial and endothelial (conjunctival and bulbar, respectively) surfaces were differentiated. The mounted tissue was coated with 12–15 nm of gold–palladium in a Polaron sputter coater and placed in an oven at 40°C overnight before being examined. The tissue surfaces were examined using a JEOL field emission scanning electron microscope operated at an accelerating voltage of 3 kV. Results were recorded both on 35 mm film and digitally. The number of cells measured for each cell type varied between 30 and 100 cells. Dimensions were compared using a two-tailed *t*-test for independent variables. Measurements of microvilli, microplicae, and microridges, and other features were performed on photographic prints using a magnifier and graticule and digital images using the Photoshop calibration tool (Version 20.0.4). At least 20 examples of each surface feature were measured (±standard deviation). Data available on request.

No attempt was made to assess the degree of shrinkage in our study. Due to the different methods of histological processing, it is expected that the same corneal features measured using scanning and transmission electron microscopy may differ slightly. However, 30–40% shrinkage is expected following fixation and resin embedding for transmission electron microscopy (Hayat 1986). Therefore, a correction factor should be applied to the data presented to give an estimate of the *in vivo* tissue and cell dimensions.

## Results

The transparent nictitating membrane of the little penguin (*E. minor*) consists of a dense stroma surrounded by epithelia on both the external (conjunctival) and internal (bulbar) surfaces (Fig. 1B–D). The nictitating membrane is approximately 139 µm thick in the periphery, where it joins the conjunctiva, defined by the presence of goblet cells on the conjunctival surface, and tapers to around 69 µm thick behind the leading tip, which consists of epithelial cells without pigment granules.

**Fig. 2:**
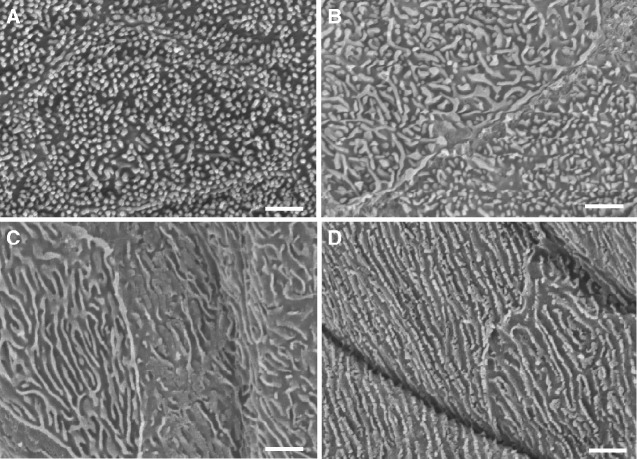
The surface ultrastructure of the nictitating conjunctival membrane in *E. minor* revealed by scanning electron microscopy. **A**) The surface of the membrane near the leading edge covered by densely-packed microvilli. **B**) Microplicae located within a short distance of the leading edge, which are short, randomly-oriented, and thicker than the microvilli. **C**) Peripheral region of the membrane surface showing numerous low microridges, which are irregular both in direction and thickness. **D**) The most peripheral region is covered by long narrower microridges of irregular thickness, which are parallel to each other and to the leading edge of the membrane. Scale bars, 1 µm.

The conjunctival surface of the membrane near the leading edge is covered by densely-packed microvilli (Fig. 2A) with a mean diameter of 120.3 ± 35.4 nm and an average length of 288.0 ± 101.0 nm. More peripherally, there are microplicae, which are short, randomly oriented projections of the epithelial surface that are thicker than the microvilli (Fig. 2B). Even further peripheral to this region, the surface has numerous low microridges, which are irregular both in direction and thickness (103 ± 35 nm) (Fig. 2C). The most peripheral region is covered by long narrower (79.0 ± 43.1 nm) microridges of irregular thickness, which are parallel to each other and to the leading edge of the membrane (Fig. 2D). These long parallel microridges appear to continue onto the conjunctiva.

**Fig. 3:**
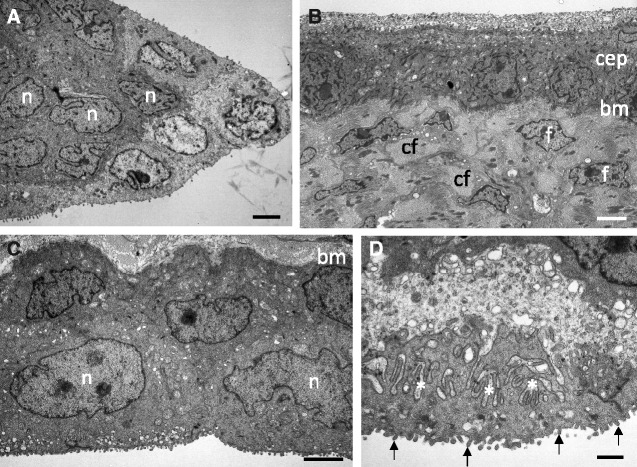
Ultrastructure of the epithelia of the nictitating membrane. **A**) The tip of the fold (marginal plait) showing stratified squamous epithelium devoid of stromal collagen fibrils. Orientation is the same as depicted in Fig. 1C. **B**) The external (conjunctival) epithelium (cep) just back from the leading edge overlying densely-packed collagen fibrils, which are somewhat randomly oriented in bundles, with scattered fibroblasts. **C**) The bulbar (internal) epithelium about mid-way between the leading edge and the periphery with three layers of squamous epithelial cells, a single basement membrane and numerous surface microvilli. **D**) Bulbar epithelial cell showing microvilli (arrows), and the complex interdigitations of the cell membranes (asterisks) between adjacent epithelial cells. bm, basement membrane; cf, bundles of collagen fibrils; f, fibroblasts; n, epithelial cell nucleus. Scale bars: 2 µm (A); 2 µm (B); 2 µm (C); and 1 µm (D).

The external (conjunctival) surface of the nictitating membrane is covered by a stratified squamous epithelium two to three cells thick near the leading edge and thickening to four to five layers of cells in the periphery. In the extreme periphery, the epithelial cells are columnar, which may represent the transition of the nictitating membrane to the conjunctiva. The leading edge and the tip of the fold (marginal plait) are composed of epithelial cells (Fig. 3A). Beneath the epithelial cells, there is a well-developed single basement membrane 32.6 ± 11.9 nm thick (Fig. 3B). Near the leading edge, the basal epithelial cell membrane is irregular and is often observed to invade the stroma (up to 285 nm), which is accompanied by a marked thickening of the basement membrane from 32.6 to around 200 nm.

The inner (bulbar) surface of the nictitating membrane, adjacent to the globe, is also covered with three layers of squamous epithelial cells, both centrally and peripherally, with a single basement membrane similar in thickness to the external epithelium (Fig. 3C, D). Scattered throughout this epithelium are a few goblet cells (Fig. 1C). The thickness of the epithelium on both the bulbar and conjunctival sides is between 8 and 11 µm, except for the peripheral area on the conjunctival side, where, instead of stratified squamous epithelium, the superficial cells are tall and columnar with a height of approximately 15 µm and the epithelial thickness is around 23 µm.

The surface of the internal (bulbar) epithelium has a few scattered microvilli near the leading edge (Fig. 2A), which become denser peripherally. The microvilli have an average diameter of 119.0 ± 24.1 nm, which is not different from the external microvilli, and a length of 234.5 ± 61.7 nm, which is less (*P* < 0.00003) than the external microvilli. There is no difference in the diameter of these microvilli between the central and peripheral regions.

**Fig. 4:**
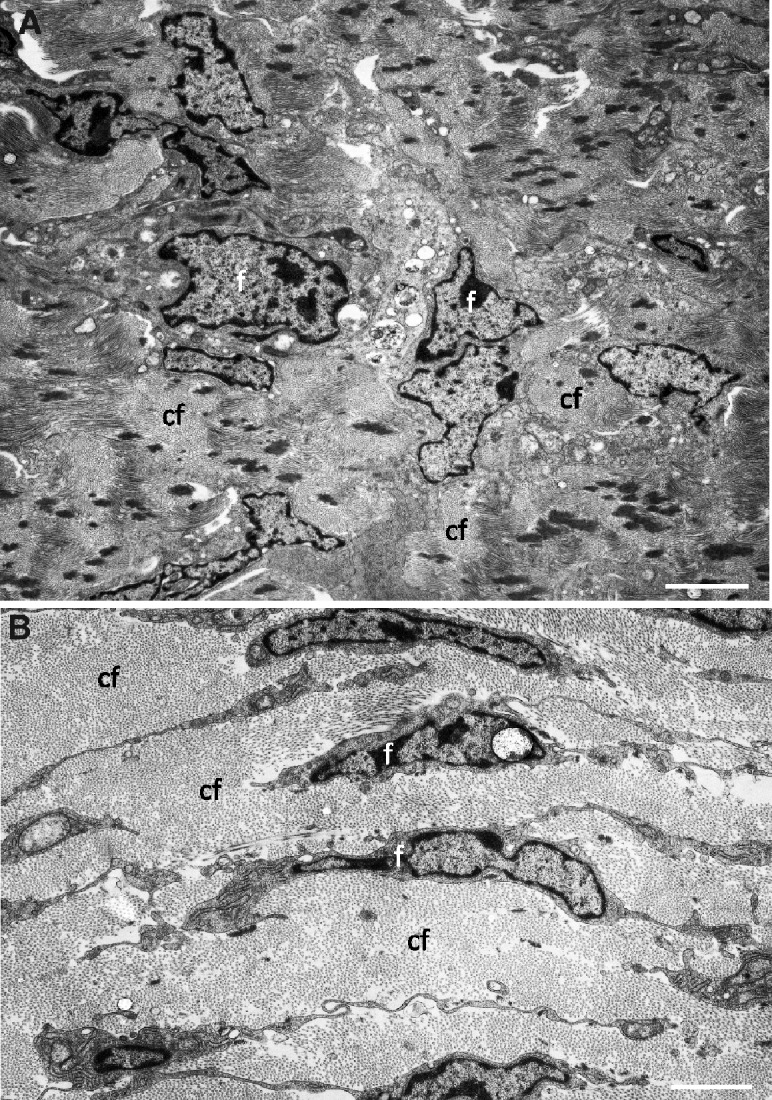
Stromal ultrastructure. **A**) A section through the stroma of the nictitating membrane immediately behind the leading edge showing densely-packed bundles of collagen fibrils randomly oriented. **B**) An area of the stroma, covering the pupil when the nictitating membrane is closed, in which the densely-packed collagen fibrils are all parallel to the surface and to the leading edge. cf, bundles of collagen fibrils; f, fibroblasts. Scale bars: 2 µm (A) and 2 µm (B).

The stroma of the nictitating membrane consists of densely-packed collagen fibrils, each with a thickness of 31.3 ± 5.8 nm and D-periodicity of 48.6 ± 4.1 nm (Fig. 4). Near the leading edge, the collagen fibrils are somewhat randomly oriented in bundles (Fig. 4A). In the center and toward the periphery, or the areas that will cover the cornea when the nictitating membrane is extended, all of the collagen fibrils are aligned in the same direction, parallel with the surface and with the leading edge (Fig. 4B). There are no divisions into lamellae. Throughout the stroma, there are scattered fibroblasts (Fig. 4), some yellow elastic fibrils and occasional blood capillaries (in contrast to the rockhopper penguin *E. crestatus*, Sivak and Glover 1986) and nerves. The nictitating membrane shows no evidence of pigmentation or a cartilaginous plate. A Harder’s gland was not included in the embedded tissue and therefore not sectioned or examined. However, a Type II Harderian gland has been shown to be present in the rockhopper penguin *E. crestatus* (Burns 1978) and the Patagonian penguin *Aptenodytes patachonica* (Reid 1835).

## Discussion

The role of the nictitating membrane is thought to be three-fold, namely to assist in the spreading of the tear film across the cornea, to remove debris from the cornea, and to act as a barrier in the protection of the cornea. For example, in ungulates, the nictitating membrane protects the cornea from grass spikes (Blogg 1980), although it is also important that some vision is maintained. Nictitating membranes have been described in many vertebrates including sharks (Poscai et al. 2017; Collin 2018), amphibians, birds, reptiles, mammals, including domestic animals such as ungulates (Stibbe 1928; Blogg 1980; Schramm et al. 1994; Schlegel et al. 2001), most non-human primates (Arao and Perkins 1968) and occurs, in a vestigial form, as the plica semilunaris in humans.

Among avian species, morphological studies of the nictitating membrane have focused on the hooded merganser (*Mergus cucullatus*) (Sivak and Glover 1986), the common mallard duck (*Anas platyrhynchos*) (Sivak and Glover 1986; Schobert et al. 2013), 10 species of owl of the families Tytonidae and Strigidae (Bohórquez Mahecha and Aparecida de Oliveira 1998), the common potoo (*Nictibius griseus*) (Bohórquez Mahecha and Aparecida de Oliveira 1998), the African black ostrich (*Struthio camelus camelus*) (Kleckowska-Nawrot et al. 2016), the barred owl (*Stix varia*) (Jochems and Phillips 2015), the bald eagle (*Haliaeetus leucocephalus*) (Schobert et al. 2013), the pigeon (*Columba domesticus*) (Kühnel and Schramm 1989), and the ostrich (*S. camelus*) (Stibbe 1928). Penguins appear to constitute the only living order of birds that are flightless and predominantly aquatic (Kooyman 1975). However, there appears to be only one study, which examines the structure of the nictitating membrane of a penguin, namely the rockhopper penguin (*E. crestatus*) (Sivak and Glover 1986).

The structure of the nictitating membrane of the little penguin *E. minor* is similar to that of other birds with a conjunctival epithelium, a connective tissue stroma, and a bulbar epithelium (or endothelium). The thickness, which tapers from 139 µm in the periphery to 69 µm at the leading edge is similar to that of the mallard duck, which has a maximum thickness of 110 µm (Sivak and Glover 1986).

The presence of microvilli on the conjunctival surface near the leading edge of the little penguin and the progression to microplicae and microridges appears to be unique. Microvilli are not present on the conjunctival surface of the membrane of the hooded merganser or the rockhopper penguin (Sivak and Glover 1986), while other authors fail to comment, possibly indicating their absence. The presence of microvilli on the leading edge of the nictitating membrane is consistent with the presence of microvilli on the cornea of most amphibian, reptilian, avian, and mammalian species (Collin and Collin 2006), while the microplicae and microridges in the peripheral region may indicate that the nictitating membrane often covers the eye when the penguin is at sea feeding, for the majority of marine species possess microridges covering their corneas (Collin and Collin 2006). The little penguin is an exception among birds, as it has short microridges over its cornea (Collin and Collin 2000).

The structure of the conjunctival epithelium of the nictitating membrane varies among birds. In the little penguin, it is two to three cells thick near the leading edge and thickens to four to five layers in the periphery, which is similar to the hooded merganser *M. cucullatus* and the rockhopper penguin *E. crestatus* (Sivak and Glover 1986) and the barred owl *S. varia* (Jochems and Phillips 2015), which also have five epithelial layers. The thickness of this epithelium also varies between 8 and 11 µm in the little penguin, 17–30 µm in the mallard duck *A. platyrhynchos*, 18 µm in the rockhopper penguin *E. crestatus*, 26 µm in the hooded merganser *M. cucullatus* (Sivak and Glover 1986), and 97.37 µm in the black ostrich *S. camelus camelus* (Kleckowska-Nawrot et al. 2016). In the frog *Rana catesbeiana*, which lives in a similar environment to the little penguin, namely, with amphibious activities, the external epithelium of the nictitating membrane is stratified and shows a well-developed keratinized layer (Lande and Zadunaisky 1970). However, in the little penguin, there is no evidence of keratinization, namely, there are no keratohyalin granules, no abnormal membranous structures, and no masses of cytoplasmic filaments (Collin et al. 1978).

The basement membrane of the conjunctival epithelium of the little penguin is single and well-defined. This is in contrast to the mallard duck *A. platyrhynchos*, the hooded merganser *M. cucullatus* and the rockhopper penguin *E. crestatus*, all of which possess a double membrane (Sivak and Glover 1986). The double membrane in *A. platyrhynchos* is also convoluted with protrusions of the basal cells into the stroma (Sivak and Glover 1986). These protrusions appear to be more marked than the small changes in the little penguin, where the protrusions into the stroma are 285 nm and only near the leading edge.

The general structure of the stroma of the nictitating membrane in vertebrates differs considerably between species and perhaps the greatest difference is the presence of thick hyaline cartilage in the majority of domestic animals (Blogg 1980), and other mammals (Stibbe 1928) including non-human primates (Arao and Perkins 1968). However, cartilage is not present in the little penguin and has not been reported in any of the birds which have been studied, nor in some amphibia, for example the frog *R. catesbeiana* (Lande and Zadunaisky 1970*).* The stroma in birds is composed of connective tissue with masses of collagen fibrils aligned in various configurations. The arrangement of these collagen fibrils determines the degree of transparency of the nictitating membrane.

The lattice theory of transparency of the cornea, which is composed almost exclusively of collagen fibrils, states that the scattered light is suppressed as a result of mutual interference. The fibrils do not scatter energy independently of one another, whereby individual scattered waves interfere destructively in all directions except that of the incident beam (Maurice 1957). For this to occur and for the tissue to be transparent, the collagen fibrils must be parallel, equal in diameter, and have their axes disposed in a lattice (Maurice 1957). The thickness (31.3 nm) of the collagen fibrils in the nictitating membrane of the little penguin is similar to that of the human cornea (27–35 nm) (Gipson 1994) and also of the frog cornea (26.5 nm) but markedly different from the thickness of collagen fibrils in the frog nictitating membrane (70 nm) (Lande and Zadunaisky 1970).

The D-periodicity of 48.6 nm for the collagen differs from the figure of 67 nm for collagen I (and II, III, IV, IX, XI, and XI) in humans (Heathcote 1994) or for corneal collagen, which has been glutaraldehyde fixed and dried, namely, 61 ± 8 nm (Jastrzebska et al. 2017). However, a mixture of collagen types II, IX, and XI in the ratio 8:1:1 gave a D-periodicity of 35–45 nm in chickens (Vaughan et al. 1988), which may indicate that several types of collagen are present in the nictitating membrane of *E. minor*. Near the leading edge, the collagen fibrils are randomly oriented in the little penguin *E. minor*, disoriented in the hooded merganser *M. cucullatus*, and loosely organized in the mallard duck *A. platyrhynchos* (Sivak and Glover 1986) indicating that vision may be poor through this region of the nictitating membrane. Back from the leading edge, all of the collagen fibrils of the little penguin nictitating membrane are tightly packed and aligned parallel with the leading edge and the surface. Hence, all of the above criteria are fulfilled, indicating that good vision should be present.

There is no stromal division into lamellae in the little penguin, although in other species, the arrangement of collagen fibrils varies greatly. In the rockhopper penguin *E. crestatus*, the stroma is described as many layers lying parallel to the surface with each at right angles to the adjacent layer, similar to the cornea (Sivak and Glover 1986). A careful examination of the figures in that paper indicates that there are approximately 100 lamellae of collagen fibrils. In the black ostrich *S. camelus camelus*, the stroma is described as a dense collagen fibril network, which implies a lack of regular structure and possibly low transparency (Kleckowska-Nawrot et al. 2016). In the hooded merganser *M. cucullatus*, the stroma is orderly, lying mainly in bundles parallel to the surface but not in sheets or lamellae, while in the mallard duck *A. platyrhynchos*, the collagen fibrils are arranged in three layers, in which two layers are roughly parallel to the surface, sandwiching the third layer in which the fibrils run perpendicular to the surface (Sivak and Glover 1986). Ultrastructurally, the mallard stroma is far less organized and within one plane of fibrils where the orientation shifts dramatically (Sivak and Glover 1986). The arrangement of collagen fibrils in the little penguin may indicate better transparency away from the leading edge and may constitute a “transparent window” as suggested by Sivak and Glover (1986). A central transparent window exists in the membrane of diving ducks (for example, the hooded merganser *M. cucullatus*, Sivak et al. 1978) and also in the rockhopper penguin *E. crestatus* and the mallard duck *A. platyrhynchos* (Sivak and Glover 1986). However, it is not evident in five representatives of the freshwater diving ducks, namely the Pochards (Tribe Aythyini) and five members of the Arctic adapted diving ducks, namely the sea ducks (Tribe Mergini) (Sivak et al. 1978).

The epithelium (sometimes reported as the endothelium) on the internal or bulbar side of the nictitating membrane of the little penguin is three cells thick compared with two in the hooded merganser *M. cucullatus*, two to four in the mallard duck *A. platyrhynchos*, and two or three cuboidal layers in the rockhopper penguin *E. crestatus* (Sivak and Glover 1986). The bulbar surface of the nictitating membrane of the little penguin is sparsely covered with microvilli (120 nm wide and 288.0 nm long). Microvilli are present on the bulbar surface in the bald eagle *H. leucocephalus* (Schobert et al. 2013) and in the rockhopper penguin *E. crestatus*, but they have been described as not numerous or pronounced (Sivak and Glover 1986). In *A. platyrhynchos*, the microvilli are thick (150–360 nm) and not uniform, while in the *M. cucullatus*, they are orderly, thick, and a uniform 1.5 µm in length (Sivak and Glover 1986). The microvilli are short and conventional (80–100 nm wide and 0.5 µm long) in the barred owl *S. varia* (Jochems and Phillips 2015). The thickness of this endothelium of the bulbar surface of the little penguin ranges from 8 to 10 µm near the leading edge to 23 µm in the periphery, compared with 2.1–3.1 µm in the rockhopper penguin *E. crestatus*, 17–30 µm in the mallard duck *A. platyrhynchos*, 26 µm in the hooded merganser *M. cucullatus*, and 74.85 µm in the black ostrich *S. camelus camelus* (Sivak and Glover 1986; Kleckowska-Nawrot et al. 2016).

In some avian species, there is a unique specialization of the bulbar surface of the nictitating membrane, called a “feather-duster” or “feathered” epithelium. In the barred owl *S. varia*, this consists of tapering cytoplasmic extensions (5 µm in width) of the epithelial cells reaching out 25 µm from the epithelial surface. In addition, narrow irregular processes called cytofilia are found among the microvilli and these extend a further 15–20 µm (Jochems and Phillips 2015). Infoldings of the plasmalemma, which fork like a brush or a small tree, and thus resemble a feather duster, have also been reported in the pigeon *Columba domestica* (Leeson 1961; Kühnel and Schramm 1989). In the bald eagle *H. leucocephalus*, the bulbar surface of the nictitating membrane also has a “feather” epithelium, although it is described as centrally-located villous projections with multiple perpendicular secondary projections producing a “fish spine” arrangement (Schobert et al. 2013). These authors found that the feather epithelium is not present in the mallard duck *A. platyrhynchos* and suggest that this is possibly because “the tear film would be under water, precluding the need for other nictitating membrane structures.” The absence of this specialization in the little penguin and the rockhopper penguin *E. crestatus* (Sivak and Glover 1986) may support this theory. In addition, the extended (1.5 µm long) microvilli described in the hooded merganser *M. cucullatus* (Sivak and Glover 1986) are more than five times the length of the microvilli of the little penguin and may represent a vestigial form of the “feather” epithelium.

The refractive indices of the nictitating membrane of six bird species were found to be similar to those of their respective corneas leading the authors to claim that the nictitating membrane is unlikely to contribute to the refraction of the eye and, when in place across the cornea, the curvature is virtually the same as that of the cornea (Sivak et al. 1978).

In conclusion, this study reveals that the little penguin *E. minor* possesses a moveable and transparent nictitating membrane similar in structure to other birds. However, the transition of microprojections (microplicae to microridges) from center to periphery, respectively, reflects a regional difference in the epithelial surface area and the level of structural support for the tear film. It appears that the “feathered” epithelium on the bulbar surface of the membrane is also restricted to non-diving birds. In contrast to the random arrangement of collagen fibrils within the leading edge of the nictitating membrane, all of the collagen fibrils are tightly-packed and aligned, thereby providing a heightened level of transparency within a central “window” allowing for clear vision in both air and water.
